# Implementing a complex hospital innovation: conceptual underpinnings, program design and implementation of a complex innovation in an international multi-site hospital trial

**DOI:** 10.1186/s12913-022-08768-8

**Published:** 2022-11-12

**Authors:** Karen Dryden-Palmer, Whitney B. Berta, Christopher S. Parshuram

**Affiliations:** 1 Institute of Health Policy, Management and Evaluation, University of Toronto, Toronto, Canada; 2 Critical Care Program, The Hospital for Sick Children, 555 University Ave, Toronto, M5G 1X8 Canada; 3 Child Health Evaluative Sciences, Research Institute, The Hospital for Sick Children, Toronto, Canada

**Keywords:** Implementation program, Implementation design, Knowledge translation, Health care innovation

## Abstract

**Background:**

Designing implementation programs that effectively integrate complex healthcare innovations into complex settings is a fundamental aspect of knowledge translation. We describe the development of a conceptually grounded implementation program for a complex healthcare innovation and its subsequent application in pediatric hospital settings.

**Methods:**

We conducted multiple case observations of the application of the Phased Reciprocal Implementation Synergy Model (PRISM) framework in the design and operationalization of an implementation program for a complex hospital wide innovation in pediatric hospital settings.

**Results:**

PRISM informed the design and delivery of 10 international hospital wide implementations of the complex innovation, BedsidePEWS. Implementation and innovation specific goals, overarching implementation program design principles, and a phased-based, customizable, and context responsive implementation program including innovation specific tools and evaluation plans emerged from the experience.

**Conclusion:**

Theoretically grounded implementation approaches customized for organizational contexts are feasible for the adoption and integration of this complex hospital-wide innovation. Attention to the fitting of the innovation to local practices, setting, organizational culture and end-user preferences can be achieved while maintaining the integrity of the innovation.

## Introduction

Implementation programs -also referred to as implementation approaches or strategies should ideally result in the adoption and sustainment of newly introduced innovations [1–3]. Despite the significant investment of time, research resources, money and collaboration involved in the creation of complex healthcare innovations, robust descriptions of effective implementation programs are limited [4–6].

Implementation of innovations intended to effect positive change in health care is notable for complexity. This complexity arises as a feature of the proposed innovation (innovation complexity), as a consequence of the implementation processes utilized for the introduction and integration of the innovation (implementation complexity), and as a characteristic of the health care setting itself and the interactions between the context, the innovation, and the selected implementation interventions [7–9]. Innovation complexity can arise when the desired practice change requires multiple steps, involves a number of stakeholders, is difficult to understand and if the innovation requires the action of groups or teams across an organization or system. Complex interventions have multiple components of change including individual behavior, technology/tools, and organizational processes (May et al., 2007). Implementation complexity can also be reflected in the degree of difficulty experienced in operationalizing activities that support use of the innovation or, the new in behaviors and process for the end-users [10, 11].

Guidance for the design of implementation programs that support the adoption of complex healthcare innovations in hospitals and in other complex healthcare settings is limited [1, 2, 12]. This is likely due to several factors that are associated with implementation processes and implementation contexts. Firstly, the processes for implementation are resource-intensive and frequently involve the allocation of significant time and organizational resources that are not often extended to evaluation of an implementation program [13]. Secondly, these programs are challenging to chronicle since the process of implementation involves navigating a myriad of complexities, including reciprocal interactions between the innovation, the setting, and the processes required for fitting the innovation and implementation interventions with the context for its use [14]. Designing and documenting implementation programs are further complicated because the innovation, implementation processes and implementation context may interact over time thereby re-shape one another. In the absence of a single best approach to implementation design, the use of a theoretically and pragmatically grounded implementation program may be helpful for navigating these dynamic challenges.

In this paper, we articulate the application of a conceptual framework for implementation and, we describe the resulting implementation program (design principles, implementation actions) for a hospital wide innovation the Bedside Paediatric Early Warning System (BedsidePEWS).

The innovation BedsidePEWS includes multiple elements of a complex healthcare innovation and constitutes considerable change from ‘usual care’ involving processes that extend beyond the systems used in the participating hospitals and in other pediatric hospital settings [11, 15–17]. The BedsidePEWS innovation involves modification to practices of clinical observation, documentation, communication and care oversight that involve all clinicians in the patient’s circle of care.

## Background

Following the creation of this novel complex healthcare innovation (BedsidePEWS), an EPOCH international cluster randomized control trial (RCT) (NCT0120831) was organized. BedsidePEWS is a team-based, hospital wide clinical system for the recognition of, and response to, hospitalized children at risk for clinical deterioration. The system requires health care providers to complete screening and scoring activities that identify patients at risk and a score-based team response matched to those risks [18]. Results of the RCT have been previously reported [15]. Beyond establishing the efficacy of BedsidePEWS, we identified the RCT as an opportunity to develop an evidence-informed approach to implementing BedsidePEWS organizational-wide, in a manner that both standardized the essential components of the innovation and was responsive to the esoteric organizational contexts, resources and the priorities of the participating hospitals and teams.

In response to this challenge, we designed and applied a conceptually grounded, evidence-based implementation framework to facilitate the design of efficient adoption and sustainment of this complex, team-based, hospital wide innovation in the differing contexts of pediatric acute hospital care. Here, implementation program refers to the coordinated collective actions, activities and processes intended to move the BedsidePEWS innovation into practice in the hospitals participating in the international RCT.

### Conceptual framework

Our review of implementation approaches for complex healthcare innovations included description of published implementation frameworks, including: PARIHS [19, 20]; Observed Knowledge Translation Application Process (OKTAP) [21]; Knowledge to Action model [22, 23]; Consolidated Framework for Implementation Research [5]; and the associated implementation elements explored in reviews, theoretical papers and model development studies [24–28]. We identified some limitations related to conceptual clarity in implementation program design and operational aspects of implementation program delivery with limited description of approaches to address the complexity for operationalizing implementation interventions [14]. The dominate approaches that surfaced assume that exposure to evidence and the innovation logically led to integration. This left unresolved practical questions about how to best account and manage the dynamic, potentially unpredictable hospital systems that refit innovations and, in turn may be influenced by the innovation over the course of an implementation [7, 25, 29–31]. We addressed these observations as we developed the Phased Reciprocal Implementation Synergy Model (PRISM) [14].

Originally published in 2020, as the ‘Tunnel Model of Implementation’, PRISM is informed by the PARIHS conceptual framework and the Observed Knowledge Translation Application Process (OKTAP) for Clinical Practice Guideline Implementation in Ontario Long Term Care Homes [14, 19, 20]. Our earlier experiences implementing organizational level innovations underscored the need for a pragmatic and theoretically consistent approach to integrating a complex innovation into existing patient care hospital systems [18]. From this review we developed PRISM while building the BedsidePEWS implementation programming due to its strength in anticipating, monitoring for and responding to, dynamic interactions between the innovation, the innovation users, the contexts, and implementation interventions across the trajectory of adoption.

The key PRISM constructs of evidence, context, and facilitation are conceptualized, as dynamic and existing on a continuum within and across implementation. Effective implementation programming should address individual behavioural change, facilitate adaptation of teamwork flow, and attend to organizational impacts of the change. PRISM structures implementation activities to optimize integration in these areas and facilitate evidence-informed implementation interventions in a phased-linked manner throughout the implementation for uptake at the individual, team, and system levels [14].

**Fig. 1:**
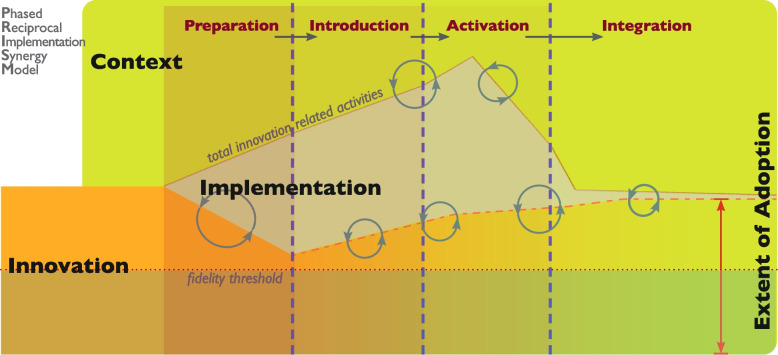
The Phased Reciprocal Implementation Synergy Model (PRISM). Legend: The processes and uptake of introducing a complex healthcare innovation into practice in a hospital context is represented in the four phases of the Phased Reciprocal Implementation Synergy Model (PRISM). The preparation phase follows the decision to implement, and implementation leaders prepare the hospital (the context) for implementation – activities may include customization or modification of the innovation to better fit the hospital context (circular arrows) and hospital. In the introduction phase clinicians and their administrators are introduced to the innovation, education is delivered, and clinical adjustments are discussed and prepared. In the activation phase the innovation moves into practice. This is associated with a peak of implementation-related activities (tan shading) that reduces as the innovation is known and understood by those using it. Effects observed in the activation phase may include modification of the innovation (lower circular arrows) and modification of the implementation activities (upper circular arrows). The integration phase is where the innovation merges and becomes a part of usual practice. The degree to which actual practice reflects the use of the innovation, as and when intended, is reflected as the extent of adoption (upper dashed line, right-side Y-axis). Below the minimum threshold of extent of adoption (lower dashed line) the innovation is unlikely to have any effect

Aligned with established frameworks (CFIR, PARIHS, KTA, OKTAP), PRISM is focused specifically on guiding implementation activities taken on after the organizational determination to adopt an innovation has been made. When organizations acknowledge a desire to implement an innovation they are committing to a change. As such, a desire to implement can be differentiated from the practicalities of organizational readiness, as PRISM focuses on inner rather than outer settings for adoption [5]. In PRISM the role of innovation end-user is not a discreet component of implementation and is viewed as an integrated component of both implementation process and context, distinguishing this model from others. The PRISM model is illustrated in Fig. 1.

## Methods

We describe the principles guiding the development of a locally relevant implementation program in multiple [10] pediatric hospital implementations in four international jurisdictions. Participating hospitals met the BedsidePEWS RCT enrollment criteria (had a Pediatric Intensive Care Unit, were anticipated to have organizational stability throughout the study and the implementation period, and were not using a severity of illness score or similar innovation prior to the introduction of BedsidePEWS) [15]. We prospectively collected observational and experiential data on the application and applicability of the framework from hospital teams across each of the organizations randomized to implement BedsidePEWS. Data was collected as part of the Hospital for Sick Children Research Ethics Board approved protocol (# 1,000,046,561) and guided by the Stage-Based Measures of Implementation Components from the National Implementation Research Network and the Ten Step Program Evaluation approach of Sridharan and Nakaima (2011) [32, 33]. Data was collected through recorded implementation meetings, feedback from organizational implementation leaders and the health care providers who are the end-users of the tool (interviews and surveys), observational site visits, and the records of the EPOCH implementation team. Verbal consent was obtained from all participants prior to data collection activities. The study team collectively analyzed the processes and activities carried out at the implementing hospitals to extrapolate the implementation program design elements and activities that influenced implementation outcomes.

### Implementation and innovation specific goals

The overarching goals of the implementation program were principle-based and aligned with the theoretical underpinnings and assumptions of PRISM [14]. Drawing on over 50 years of combined clinical education and practice experience, the study team (implementation leaders [2], an expert educator [1], and education coordinator [1]), explored past implementation challenges and successes with the BedsidePEWS innovation and distilled these with the current published evidence guiding implementation. The resulting implementation program goals included: 1] integrate the innovation into the existing organizational culture and learning/unlearning systems; 2] actively support practice integration through social mechanisms and 3] allow for iterative feedback across the scope of the implementation.

Specifically, for the implementation of the BedsidePEWS, four innovation specific objectives were developed: 1] ensure technical fidelity of the BedsidePEWS innovation; 2] embed the pragmatic components of the BedsidePEWS into routine hospital/team practices; 3] enable ongoing formative and summative assessments of implementation processes—including the level of adoption achieved, and 4] facilitate responses to any threats to innovation uptake emerging during the implementation interval.

## Results

In this section we describe the implementation design principles applied to BedsidePEWS implementation, the operational strategies observed, the innovation-specific tools developed, and implementation program outcomes across the participating hospital sites.

### Overarching design principles

We identified several theoretical design principles aligned with selected program theory and applied to the implementation program design. The following five principles enabled a focused and comprehensive approach to implementation planning in each PRISM phase and at each participating organization: 1] flexibility and responsiveness of implementation actions to organizational context; 2] thoughtful and measured customization of the innovation and implementation activities; 3] optimized end-user engagement; 4] intentional integration with existing organizational process and 5] leveraging social factors. Each principle was observed to influence implementation programing at each hospital and were key in the development of a context responsive implementation program at each site.

#### Make implementation planning and activities flexible and responsive

The first design principal of flexible and responsive implementation planning attends to the dynamic relationship between the innovation, the organization, and the implementation processes [14]. The goal of responsive planning is to improve the ‘fit’ of the implementation with the local environment and facilitate organizationally established and familiar approaches to implementation. The flexibility created in planning responsive actions across the implementation program phases enabled space for ‘in process’ adaptations and allowed for nimble responses to the anticipated and unanticipated impacts of both the innovation and the planned implementation activities. This responsive facilitation of new practice behaviours and early recognition and management of potential threats to the desired change(s) associated to BedsidePEWS enhanced process efficiency. Intentionally embedding responsiveness allowed implementers to anticipate and monitor for operational signals and adapt implementation activities e.g., aligning timelines with organizational priorities to account for competing projects/demands. In effect, implementation teams and organizational leaders ‘expected’ to adapt the implementation plan and activities throughout the implementation program interval. There was no ‘set it and forget it’ mind set. Flexibility and responsiveness were achieved through short interval implementation evaluations (weekly), focused routine inquiry to surface anticipated and unanticipated impacts (standardized in implementation meetings), and short loop responses (within days) to emerging issues.

#### Customization of the innovation

The second principle of customization of the innovation refers to both the intentional and unintentional modifications that occur because of its introduction and or the implementation processes by which it is moved into the context. Local modification(s) should be carried out with attention to preserving innovation fidelity, in that the core components of the innovation be protected from revision that would take away from the impact of the innovation. Modifications should be anticipated by implementing hospital teams to both enhance implementation efficiency and reduce innovation erosion.

Unregulated customization risks altering the innovation to the point of limiting the benefits or disrupting the impacts of the change. Preservation of innovation core elements was maintained through clear articulation of the content/components of the innovation from the start of implementation and sustained across all implementation activities and stages. For this innovation these core components were identified as the scientifically validated BedsidePEWS score, the stepwise escalation of care in response to the patient score and the frequency of patient screening. These components were identified as non modifiable and how they would interface with existing care process were collaboratively explored by the local implementation team, guided by the study team, in early implementation preparation. Adaptation of the remaining components of the innovation and the implementation program were proactively evaluated for adaptation to the specific setting and user groups at each site, for example, local vernacular (including translation to preferred practice language), team roles, organizational resources, educational delivery methods and communication processes. This was accomplished by actively moderating these activities and the oversite of the external expert team to provide local implementation leaders with anticipatory and responsive support to any emerging threats to the innovation.

#### Optimized end-user engagement in implementation

The third principle arose from the observation that the innovation, and how it is applied in practice, are shaped by local culture including end-user expectations, priorities and values related to early warning systems. Participatory implementation strategies that engaged end-user s, catalyzed individual, team, and organizational ‘ownership’ of the innovation [3, 6]. Strategies included representation from all end-user groups (clinical nurses, physicians, educators, allied health) on implementation planning and leadership committees, actively seeking feedback about the design of the innovation, end-user preferences for education and awareness programing and leveraging end-user experiences and stories throughout the implementation period. End-user participation in planning and operationalizing customization of the innovation and the implementation activities required end-user to work in a proactive way and accept ownership of the implementation outcomes. This principle and the resulting strategies were noted to be equally effective in ‘top down’ leadership-driven hospital implementation programs as well as grass roots initiatives primarily driven by the end-user themselves.

Participating hospitals integrated end-user as members of the implementation planning team and often were situated in key implementation roles, for example, clinical educators to customize and deliver education, clinical experts from various disciplines contributing as champions. End -user participation was typically introduced as soon as the organization was enrolled to implement. End-user perspectives about the implementation were discussed at planning meetings and end-user contributed to implementation program goal setting and implementation evaluation strategies were sought. End-user input was solicited through organizational surveys, focus groups and testing the BedsidePEWS with prospective historical case records and simulated patient scenarios.

#### Integrate implementation activities with existing team and organizational processes

The fourth principle of intentional leveraging of existing team and organizational processes, facilitated ‘normalizing’ of any innovation associated activities, supported continuity with existing organizational processes and helped to increase alignment of the BedsidePEWS within the organization’s existing practice culture [11]. A consequence of this design choice is the need to know, understand and access the specific processes that can support uptake. Collaborative planning between the external experts and local teams is essential to achieving this. Early assessment and activation of existing educational and marketing platforms that align with BedsidePEWS content and using existing proven education approaches for end-user training are examples of how this principle was applied in the implementing hospitals.

#### Leverage existing social processes to support implementation

This fifth design principle acknowledges the important impact of social processes such as role modelling and opinion leader support within an organization to galvanize support for the desired change(s) [34]. Explicit exploration and leveraging of the supportive social process specific to each implementation hospital provided a vital connection between implementation leaders and end-user, facilitated communication about the innovation and the implementation process, legitimized the innovation, and assisted end-user to ‘let go’ (unlearning) of existing practices in favour of the new ‘preferred’ behaviours [35]. Application of this principle also required attention to, and mitigation of, social processes that were working against or eroding acceptance of the innovation, for example an influential team member encouraging rejection of the innovation. Operationalizing this design principle required local knowledge of these processes and the identification of key people influencers within the targeted end-user groups. Providing an open platform for the voices of key individuals, openly embracing, and addressing concerns directly and developing implementation roles to support social pathways were examples of how implementers aligned social mechanism of influence with the implementation program goals. Hospital teams designed internal communities using peer coaches and clinical leaders to support the clinical application of BedsidePEWS and established innovation specific pathways for communication about their own experiences using this innovation. Social processes external to the organization were also leveraged to facilitate effective implementation. The formation of a community of practice (COP) linking all implementing hospitals involved in the project allowed individual hospital teams to share and explore with each other ways to enact social facilitation [5].

### Operational design strategies

Hospital teams preferred the phased approach to structuring the implementation program with the goal of optimized efficiency in dynamic hospital environments. Phased implementation permitted thoughtful iterative and cumulative evaluation of smaller scale implementation activities in the units across a given organization and provided a means for end-user to teach and lead one another from within. The phases provided structure to implementation progression, afforded interval opportunities for review and timely communication of progress across the organization and aligned activities to focus on the goals of each phase. The phases facilitated stepwise knowledge transfer across the organization. The emergence of the foci, goals and subsequent activities in each phase were consistently observed across participating sites.

Specific operational sub-goals linking the theoretical underpinnings of the implementation program to the operation aspects as applied to BedsidePEWS are articulated within each phase of the implementation program in Table 1. Table 2 outlines implementation activities observed in each of implementation phases.

**Table 1:** Phases of PRISM, foci, theoretical underpinnings, and the associated operational goals

Phase	Preparation	Introduction	Activation	Integration
Foci	Anticipatory Planning Customization	Hospital wide awareness End-user and stakeholder engagement Adaptation of implementation interventions/actions Formative evaluation	Innovation integration to practice setting/systems Facilitation of innovation evaluation/revision	Normalization Transition Sustainment
Theoretical under-pinning	Enhancing innovation fit to context will improve acceptance and adherence to the desired change(s) [35] Improving organizational readiness for change can improve implementation outcomes [36]	Skills to operationalize innovation must be built or refreshed close to the time of use [18]	Leveraging social mechanisms of influence improved implantation outcomes [34]	Active integration of the innovation into organizational reservoirs of knowledge will facilitate sustainment of the innovation after implementation [37]
Goal(s)	1] Explore the organizational preferences and resources and anticipate factors that may influence success across the implementation continuum 2] Determine organizational readiness for change 3] Customization of education, marketing/dissemination strategies, evaluation measures, and sustainment planning to optimize innovation fit and mitigate factors impacting adoption	1] introduce innovation to stakeholders Maximize stakeholder engagement 2] Trial/retrial implementation interventions- Refine implementation plan 3] Refine fit of innovation 4] Integrate sustainment activities	1] Launch innovation into practice 2] Ongoing evaluation of impacts 3] active support of support of new practices/ expectations 4] Refine innovation in relation to context of use 5] Reinforce the protected core elements of the innovation	1] Normalize new practices/behaviours Optimize links with within and between practice communities 2]Consolidate sustainment interventions 3] Embed the innovation into administrative and clinical routines,

**Table 2:** PRISM phase- based implementation activities

Phase	Preparation	Introduction	Activation	Integration
Activities **(activities may extend across multiple phases)**	Develop responsive implementation plan Weekly consultation with external implementation experts* Establish local implementation team Relationship building with external implementation team* Readiness assessment (SWOT analysis, environmental scan, Stakeholder interviews/canvassing) Determine organizational implementation (timeline, interventions & evaluation) Customization of the innovation, education for the introduction stage, and education materials/prompts. For example, adapting documentation forms, communication processes to fit each organization* Develop marketing, awareness, and communication plans* Obtain organizational/ decision-maker/clinical leadership endorsements*	Launch the marketing, awareness, and communication plans Engage end-user in initial innovations piloting and testing (simulation, case reviews and debriefing) Disseminate new practice expectations associated with the innovation Refine innovation fit based on initial feedback and evaluation. * Pilot/refine implementation interventions. * Provide stakeholder relevant education/training* Launch practice integration support strategies*	Audit and feedback performance to end -users and decision-makers. * Embed roles and social connections to reinforcement of new practices/behaviors (champions, coaches, team competition, link participating organizations to achieve a broad community of practice related to the innovation) * Observe for and address unanticipated impacts of change Continued refinement of innovation for fit. * Ongoing multi-model Education/training/skills refinement. *	Activate sustainment plan targeting each organization’s reservoirs of sustained change Decrease frequency of consultation with external implementation team Trend relevant outcomes: adherence trends/care impacts- or innovation relevant data. And integrate into organizational performance indicators, Embed innovation into existing routines, organizational metrics, roles, or organizational informational networks

### Preparation phase

Following the organizational decision to adopt, and randomization to implement, the implementation program focus is on establishing readiness to use the innovation, customization of the innovation and the implementation activities and establish readiness to use the innovation. This was lead in a collaborative two-team framework with (i) an external team of innovation content experts and (ii) an internal/local team of organizational experts. This approach provided external stewardship of the innovation and support for the adaptations that occurred across the scope of the implementation. The external team served as content experts about the innovation, bringing broad experience with the innovation to facilitate solution-building for local challenges over the course of implementation. The external expert group was noted to enable facilitation of the customization of the innovation while preserving the core components and provided the cumulative implementation knowledge and experiences of previous implementations to be available to the local planning team.

The local core implementation team contributed local system and resource knowledge to implementation planning and capitalized on existing relationships and connections within the organization. The required characteristics, skill sets and scope for local team members was determined by each implementing hospital and often included physicians, administrators, quality leaders, front-line providers, researchers, and educators. The local implementation team within each organization interacted with other teams in their setting, and shared information with the external content experts that supported alignment of the BedsidePEWS with local processes and resources. Together external and local implementation teams developed implementation roles specific to each organization. The local leadership team determined how each role operationalized within the existing organizational structure and who in the organization would best fill the roles. End-users were integrated into the activities of this phase through membership and participation as primary implementation planners or as consultants to the local implementation team. Table 3 describes the four different implementation roles that emerged, the associated responsibilities, organizational positions filing these roles, the phases, and domains where each role was influential.

**Table 3:** Implementation roles

Role	Implementation Responsibilities	Organizational positions	PRISM phase	Domains of influence
Primary implementation planners	Organizational level decision-making and stewardship of implementation processes Oversite of customization of implementation program materials/ activities/timing/evaluation Manage organizational level barriers to optimization the implementation interventions Design education, select marketing and dissemination activities Oversight of material production and distribution, respond to challenges emerging during implementation Design sustainment activities	Key decision makers Local unit/team leaders Research and education leaders End-user/Frontline clinicians	Preparation Introduction Activation Integration	Organizational level Resource allocation Project oversite
Secondary implementation operators	Deliver implementation interventions Monitor implementation activities/impacts and feedback to implementation teams Participate in ongoing customization and integration of innovation Organizational dissemination about the innovation	Educators Selected Frontline clinicians/end-user identified by the local primary team as influencers in the end-user communities	Introduction Activation Integration	Team and individual level Practice integration Role model/Early Adopter
Tertiary implementation facilitators	Disseminate information about the innovation to individuals and teams Support integration of the innovation at point of care	Frontline clinicians who are positioned to facilitate use/application of the innovation Champions	Activation Integration	Team and individual level Facilitate and apply innovation
End-user *Being an end-user can be inclusive with other implementation roles	Participate in Implementation activities Feedback impacts and outcomes of the innovation Implementation design/planning advisor	Any team member or frontline clinician who will use the innovation	Preparation Introduction Activation Integration	Team and individual level

In the Preparation phase, implementation team meetings focused on the customization of implementation materials, designing organizationally relevant implementation interventions, establishing timelines, modes/methods for education delivery, and evaluation measures such that activities were meaningful to the organization and to end-users.

Timelines for each phase were determined collaboratively between the local and external implementation teams. The local team played a central role ensuring implementation activities complemented other planned hospital activities, are aligned with organizational priorities, and helped to inform and engage other hospital leaders in decision-making and problem solving.

A collaborative ‘experience with change’ and environmental scan were key activities in the Preparation phase. A Strength, Weaknesses, Opportunities and Threats (SWOT) matrix structured these assessments [38]. The SWOT framework explored past experiences with organizational-wide change and surfaced organizational strengths for consideration of how the identified strengths might be leveraged in the context of the current implementation. Known gaps or areas of weakness were then addressed and mitigated in the implementation planning process. Alignment of the implementation plan to the organization’s priorities was pursued as part of this assessment along with exploration of the potential unexpected impacts or threats to sustained adoption and mitigation strategies developed [39].

Elements of readiness for implementing were assessed at the organizational level and included articulation of the organization’s motivation to implement the innovation (a response to a clinical situation or event, scientific exploration, as a duty to stakeholders, responding to industry pressures, person driven innovation), organizational decision-maker commitment to the change, identification of local mechanisms to engage end-user in the implementation process and pragmatic preparation in terms of resources and organizational climate [36]. A purposeful pre-emptive review of the unique organizational context, prior to introducing the innovation, with attention paid to the interests of all stakeholder groups was an important part of implementation planning and achieving contextual fit.

The local and external teams developed implementation specific communication plans, for example structure weekly implementation meetings. Program timelines created during the Preparation phase provided a road map of explicit milestone dates/intervals, for example, ‘go live date(s) that were set across an organization or as a series of timelines attached to interorganizational units or teams.

The external and local implementation teams collaboratively developed customized education materials and practice prompts and built safeguards to preserve fidelity of the innovation (for example audit criteria and adherence goals). Organizationally relevant performance measures were set, marketing and dissemination activities developed and planning for sustainment was addressed during in this phase. The tasks in this phase focused mainly on planning that leverages local expertise and existing organizational practices, whist integrating the implementation expertise of the external team. This was accomplished through regular (minimum of weekly) team meetings and frequent email exchanges between the organizational implementation team and the external team for the duration of the implementation program.

### Introduction phase

The Introduction phase focused on activity supporting end-user exposure to the innovation, organization-level endorsement of the innovation, the associated implementation program, and foreshadowing of anticipated changes that users might experience. Specific actions included town hall meetings, grand rounds, activation of the hospital’s communication networks, awareness campaigns with posters and emails to stakeholder groups. Gestures of support from organizational leadership and selected endorsement from influential individuals was an important component of the initial exposure end-users had to the innovation. Organizational leader support took the form of letters, public statements of endorsement, personal participation in implementation activities and or securing access to resources for the implementation team and end-user for example providing educational time.

Activation of the planned implementation activities was the main implementation output of this phase. Implementation teams and selected primary implementers reviewed the implementation interventions and activities developed in the Preparation phase and selected those of relevance to their practice setting and current context. The interventions were adapted to achieve local ‘fit’ in this phase to support compatibility with existing processes and norms. End-user involvement was encouraged and, as per the design principles, attention was paid to utilizing the social processes that supported innovation use in this phase.

Tertiary implementation facilitators supported awareness and learning about the innovation as well as provided a conduit for feedback to implementation leaders about progress including expected and unexpected impacts. Customizations achieved in the Preparation phase were trialed and revised as needed in an ongoing way. Introduction phase interventions and activities included: short loop communication processes between end-user and implementation leaders (message hot lines, comment boxes, canvasing for feedback), initiating social mechanisms of influence within teams and units (champions, peer coaches), marketing of the rationale for the change (posters, web page banners), and disseminating the new practice expectations (education and team meetings). In this phase, implementation interventions, evaluation strategies and facilitated learning processes were incorporated in a responsive fashion with ongoing refinements occurring into the activation stage. This ongoing communication was enabled by regular, frequent scheduled meetings between stakeholder groups and local implementation leaders that continued until the implementation program was completed.

Local and external implementation teams continued to meet at regular intervals, to evaluate input from end-users, revise the implementation plan, and mitigate/manage unintended consequences and to identify emerging challenges related to the evolving implementation. Connecting end-users and implementation leadership with other successful implementing organizations in this stage was helpful for sharing experiences implementing BedsidePEWS, and for solution building to emerging implementation program challenges. The external implementation team acted as a link between implementing organizations to achieve this. This phase advanced to the activation phase at the completion of planned pilot trials, or ‘run-in’ exercises when a critical mass of end-users are prepared to apply the innovation in their practice.

### Activation phase

The Activation phase centred on the application of the innovation in the setting. This phase included ongoing assessment of the innovation and its impact. Solicitation of end-user feedback (interviews/survey), team level impacts (case debriefings) system level changes (serial environmental scans) and innovation outcomes measures (patient level quality indicators) were all sources of feedback utilized by implementing organizations. This was actioned collaboratively with external implementation team, coupled with primary and secondary implementers within the organization. The continued customization of the innovation respecting the established core parameters required active integration of end-user feedback as well as intentional integration of the innovation associated process (clinical escalation pathways for example) with existing familiar workflows. Focus here was on making the innovation operational in the various contexts across the organization and supporting technical as well as social integration of the innovation. Secondary, tertiary, and end-users-maintained vigilance for the required innovation specific behaviours and continued to guide point of care application to preserve the fixed components of the innovation. This approach served to enhance familiarity and fit of the innovation with existing practices, language, and supported end-user engagement with process.

In the Activation phase, end-user engagement processes included input into formal and informal evaluation of the innovation and exploration of the impacts of implementation interventions. This feedback was solicited in person, by survey, in team meetings, via rounding activities, one-on-one discussion, case reviews and simulation sessions. The resulting feedback directly informed the ongoing modifications to implementation activities, educational programing, practice materials, and supportive integration activities. Reviewing this feedback also emerged as part of regularly scheduled external and local implementation team meetings during this phase. Audits of innovation-relevant practices and adherence measures for core innovation components complemented informal practice reviews and provided opportunities for targeted responses to any threats of innovation erosion. End-user and decision-maker surveys and informal interactions with implementation champions and coaches help to build situational awareness related to implementation progress, identify any threats, and facilitate the dissemination of successes achieved to date.

Scheduling and oversight of education delivery was organized and administered by the local implementation team. Interval evaluation of knowledge uptake and measures of end-user integration of the innovation was addressed in this phase. Strategies to facilitate learning and unlearning, as designed in the Preparation phase, were launched at this time, and were customized to the learning norms of the individual professional groups involved. Learning strategies included onboarding of new staff as well as re-fresher education/support, and point-of-care education. Multiple modalities for education and learning were created within each hospital including didactic, self-learning packages, online training, and competencies approaches. Leveraging social mechanisms for change, for example, collegial competitive reporting (setting organizationally relevant performance goals and contrasting performance between teams/units) supported team level change. Strategies facilitating both new behaviours and unlearning of old practices/behaviours included: simulation; case reviews and debriefs; public acknowledgements of team or individuals leading practice; and one-on-one coaching. These same strategies were anticipated to support sustainment of the innovation and mitigate the re-emergence of prior practices in anticipation of the implementation program completion [37].

### Integration phase

Integration is the fourth phase where the innovation is solidified as “usual practice” and set up for sustainment in the organization [11]. In the Integration phase activation activities continued. Roles associated with the implementation program were reorganized such that innovation-specific responsibilities were taken up as part of existing positions, or alternatively, permanent innovation oversight roles were created. For example, surveillance for innovation adherence might continue to be led by the hospital quality improvement team or a specific role may be developed for this task. In this phase, implementation planning focused on facilitating the integration of the innovation into organizational reservoirs of knowledge [37]. Ways in which the innovation was taken up in reservoirs included integration of the innovation into in the organization’s onboarding processes, embedding the innovation in organizational policy and procedure, and incorporation of performance measures associated to the innovation at the patient, team and system levels, as appropriate. Implementation program evaluation measures were modified in this stage to be incorporated with ongoing hospital evaluation plans and priorities. Implementation teams in this phase capitalized on opportunities to solidify the social mechanism(s) that supported the implementation in the activation phase, for example formalizing champion roles or continued participation in communities of practice [34, 40].

Sustainment planning was a dominant component of the Integration phase. Sustainment planning by local implementation teams included building and/or activating existing reservoirs of organizational learning to support ongoing adoption [37]. Examples of sustainment interventions included: sharing of ‘good outcomes’ associated to the innovation; highlighting case examples of effective application of the innovation; creating platforms for sharing experiences related to the innovation; integration with established organizational communication pathways (standardized documentation and agenda items for team meetings). Establishing sustainment activities, concurrent with the gradual transition to local team leadership for ongoing ownership of the new practice(s), marked the end of the integration phase and the implementation program.

### Implementation materials

Implementation materials developed for this program consisted of a core set of innovation-specific materials provided to the local team by the external implementation team. These materials were available for modification across differing hospital contexts. New materials were also iteratively developed by the local implementation teams as well. For the innovation of BedsidePEWS the core material set included multi-model core education generalised for all end-users, catalogues of training cases and marketing materials (for end-user, decision makers, patients/families, and the general public). Marketing materials were available for refinement by local teams (for example posters, clipboards, posters, pocket cards). Materials were intentionally flexible for delivery in a variety of settings. The collaborative (external and local team) modification of core implementation materials to fit local needs was undertaken in the Preparation phase by primary implementation team planners in collaboration with secondary implementation operators, tertiary implementation facilitators and end-users of the innovation. Table 4 provides an overview of the BedsidePEWS specific implementation materials that were developed in each phase and identifies the fixed and customizable elements of each.

**Table 4:** Core implementation materials

Materials	Type	Target Audience	Fixed elements	Customizable elements
Pocket cards Clip boards Rulers Posters	Prompt	End-user	Content	Language Formatting Distribution methods
Tip sheet ‘Frequently asked questions’ sheet Scenario/simulation library Technical manual	Education	End-user Primary Secondary Tertiary	Content Learning outcome measures	Language Formatting Distribution methods Format Timing Delivery modalities
Education workshops (lesson plans, slide decks and teaching materials) Web-based self-directed learning module Self-test	Education	End-user	Content Learning outcome measures (measures customized for learner groups; discipline, learner level or interests)	Language Formatting Distribution methods Format Timing Delivery modalities
Briefing note Plain language pamphlet Health care provider pamphlet Introductory letter-health care providers	Health care provider Awareness	End-user Primary Secondary Tertiary	Standardized content	Language Formatting Distribution methods
Introductory letter-families Information Pamphlet	PublicAwareness	Clients/family Public	Standardized content	Language Formatting Distribution methods
Web site	Community building	Within and between organizations	Content curated by BedsidePEWS team	
Annual innovation specific academic meetings	Community building	Health care community at large	Collaboratively designed and delivered by the community of practice/external experts	

### Implementation evaluation

Implementation program evaluation in the Preparation, Introduction, and Activation phases was primarily formative with the information feeding back directly into the iterative components of the implementation programming. Utilizing short loop evaluation cycles and vetting of the customized materials/products across the Planning, Introductory phases allowed for close monitoring of innovation integrity and fit within the multiple contexts across an organization, and refinement of the implementation methods themselves. These formative measures included implementation team functioning, post-education learner feedback, end-user perceptions of the quality of their preparation to use the innovation, utility and adherence with the innovation associated behaviors and practices. End-user adherence to the requisite practice behaviours was audited every week to two weeks, and the results discussed at the routine implementation meetings. Adherence data along with clinical event-based case reviews and secondary implementation role observation informed recommendations for ongoing revision and refinement of the implementation program.

In the Integration phase, evaluation activities became more summative and focused on the innovation specific patient, team, and system level impacts as well as the outcomes of the implementation program itself. Measuring these impacts complemented and extended the earlier assessments of the innovations’ value and were important to inform future implementation projects and extensions of the innovation within the organization. Table 5 outlines the evaluation approaches and examples of potential evaluation questions associated with each of the PRISM phases.

**Table 5:** PRISM evaluation approaches

Phase	Planning	Introduction	Activation	Integration
Approach	Anticipatory Formative	Formative	Formative	Summative Monitoring sustainment
Domains	Motivation Needs-assessment Resource review	Customization Awareness	Innovation refinement Implementation activity refinement Stakeholder impact Unexpected outcomes Innovation associated outcomes	Adherence Quality Consistency
Organization Level^a^	Structures	Processes	Processes Impacts	Outcomes (patient, system, organizational
Process level^b^	Trial and test	Reaction Learning	Reaction Learning Transfer	Transfer Results (individual, team)
Potential questions	What factors may influence implementation success? What resources (structural, human, monetary, operational, time…) are needed? Will the innovation ‘fit’ the targeted setting(s)? What needs can we anticipate and address?	Are end-users prepared to use the innovation? Is awareness adequate across the organization? Are all stakeholders heard/represented in process? Does the innovation function as anticipated? What is the impact of implementation materials resources and activities? What factors might be inhibiting or facilitating uptake of new behaviors or practices Are there adequate support to unlearn old practices? What are the motivations/tone of the change?	Has the innovation achieved desired impacts? Are there areas that require focused implementation efforts? What are the adherence levels? Is fidelity of the innovation preserved?	What are the unanticipated impacts of the change? Are there threats to sustainment? Has the innovation penetrated the knowledge reservoirs of the organization? What is the extent of adoption of the innovation?

^a^Organizational level evaluation approach was informed by Donabedian’s Model of Health Care Quality [41]

^b^Implementation process level evaluation approach was informed by Kirkpatrick’s Levels of evaluation [42]

## Discussion

Five key lessons emerged from our experience with the implementation of BedsidePEWS that may guide future use of this implementation program design and the approach that we describe here.

### Lesson 1: Grounding implementation approach in theory assists conceptually & practically

First, this report of a theoretically grounded implementation approach for a single complex innovation spanning multiple diverse hospital organizations offers a practical approach to apply theoretically grounded implementation design. This approach may be applied to other complex hospital-wide healthcare innovations.

The generation of foundational design principles and the PRISM operational framework provide a functional means to plan for, apply, evaluate, and modify innovation-specific implementation processes and actions. The use of a conceptual framework enabled explicit description and evaluation of individual and cumulative impacts of the implementation program interventions. Mapping of implementation phases specific goals to the program interventions in each organization prevented duplication of less effective implementation strategies in new settings. This also enabled capacity building related to implementation within the individual organizations as well as between organizations.

### Lesson 2: A phased approach to implementation offers several advantages

We describe the potential advantages of phased approaches in the operationalization of implementation programs. Phased implementation approaches enabled: 1] the ability to ‘pilot’ test the innovation in-situ. This can permit identification and modification of factors impacting the innovation’s introduction and fit and any unexpected impacts on other organizational activities; 2] the ability to tailor implementation activities to specific needs and timelines for various end- user groups; 3] pacing of the implementation program to align with organizational goals, resources and priorities; 4] strategic leveraging of facilitative relationships between units and within/between organizations, including competitive motivations, role modeling and building connected communities of practice focused on the innovation; 5] supported reflexivity, early recognition and revision of activities that are not meeting implementation objectives or are contributing to fidelity/erosion issues; 6] preemptive mitigation of complexity of the innovation and the environment into which it is applied.

### Lesson 3: Consideration of innovation “fit” can improve implementation processes

We demonstrated that the pragmatic application of a flexible approach during the implementation-adoption continuum increased fit of the innovation and implementation programing to each hospital setting. Design of the core implementation materials and tools enabled modification that preserved essential innovation specific elements, enhanced fit and was central to the success of the implementation program. Planning for customization as a key activity in implementation provided for alignment of the innovation to differing cultures of practice and building in flexibility in the materials and implementation interventions (for example audit and feedback approaches) plays to the existing organizational strengths.

### Lesson 4: Consider sustainment concurrent with implementation

Intentional sustainment planning ensures the penetration of organizationally relevant reservoirs of knowledge [37]. Fitting the innovation into educational, information, social and procedural spaces in the organization increased the compatibility of an innovation across the organization and supported normalization of the innovation into routine practice. Approaches to achieve this varied between implementing teams, disciplines, and organizations. Local as well as external implementation expertise are crucial to uncover and leverage the most impactful sustainment activities and knowledge reservoirs.

### Lesson 5: End-user engagement benefits planning & operationalization

Embracing end-user participation in implementation planning and operationalization across the continuum of the PRISM phases enhances the transparency of the process and strengthens end-user ownership and organizational level problem- solving**.**

## Limitations

There are limitations to the generalizability and representativeness of the approaches described in this paper. Our approach is dependent upon revising for ‘fit’ on a continual basis. This requires the time and expertise of local teams who are assumed to know, or have access to, knowledge of local culture, learning reservoirs and organizational resources. Incomplete or misinformation in these areas may undermine the success of implementation programming and efforts.

This implementation approach requires high levels of organizational attention and support, including allocation of discretionary organizational resources both tangible and human. Without this level of support, innovations may be vulnerable to competition from other organizational priorities and/or the demands on implementation leaders who carry other roles and duties. A balanced approach is required in terms of engagement of leadership, frontline, education specialists and research/quality improvement expertise.

Our conceptually grounded implementation program design is untested beyond the BedsidePEWS project. Attention was paid to implementation approaches that preserve the integrity of the innovation as the customization process can risk dilution of the innovation benefits unless carefully curated and evaluated across the implementation process. This approach may not be transferable to other innovations or settings.

Evidence of the effectiveness of this administered program is predominantly indirect. The impacts of specific implementation interventions and descriptions of the interactions between BedsidePEWS, implementation processes and the differing contexts are not directly explored in this paper. The randomized trial of this innovation’s overall impact demonstrated improved processes and timeliness of care but did not achieve the innovation’s primary measure of improved patient mortality [15]. Further evaluation of the relationships between the nature of implementation, the interactions between innovation, implementation and context and the extent of innovation adoption will provide additional evidence about the overall effectiveness of the approach taken to developing a customizable implementation program for use in a diverse range of hospitals. Furthermore, application of this approach to other complex healthcare innovations will provide additional evidence of the validity and utility of the design approach taken here.

## Conclusions

Successful adoption and sustainment of an innovation in the context of complex health care organizations requires behavioral, conceptual, and cultural changes by the participating providers, teams, and organizations. Therefore, implementation design must address the skills and pragmatic requirements for using the innovation as well as the relational and contextual issues that influence change in healthcare settings. We believe that describing and evaluating implementation strategies for complex healthcare interventions should be a routine part of effective knowledge translation practices.

We have illustrated a conceptually grounded and locally customized implementation program that is feasible for the adoption of complex hospital wide innovations. Implementation programs differ between organizations and within organizations, one size does not fit all. Creation of implementation programs based on considered design principles, integrating implementation knowledge leaders, engaging local organizational and practice experts are a key precursors of successful innovation adoption. Attention to the fitting of an innovation to local practices, the setting, organizational culture, and end-user preferences can be achieved while maintaining the fidelity of the innovation. Future implementation research should prospectively explore innovation fidelity and process of sustainment emerging from implementation activities and sustainment measures. Articulation of detailed description of the implementation strategies for complex healthcare interventions are foundational for effective knowledge translation and enduring change.

## Data Availability

All data generated or analysed during this study are included in this published article.

## Abbreviations

BedsidePEWS: Bedside Paediatric Early Warning System
PRISM: Phased Reciprocal Implementation Synergy Model
RCT: Randomized Control Trial
CFIR: Consolidated Framework for Implementation Research
SWOT: Strength, Weaknesses, Opportunities and Threats
COP: Communities of Practice
